# Development of a Two-Dimensional Model for Predicting Transdermal Permeation with the Follicular Pathway: Demonstration with a Caffeine Study

**DOI:** 10.1007/s11095-017-2209-0

**Published:** 2017-06-28

**Authors:** Panayiotis Kattou, Guoping Lian, Stephen Glavin, Ian Sorrell, Tao Chen

**Affiliations:** 1 Department of Chemical and Process Engineering, University of Surrey, Guildford, GU2 7XH UK; 2 Unilever, Colworth Science Park, Sharnbrook, Bedfordshire, MK44 1LQ UK

**Keywords:** bioavailability, diffusion, in silico modelling, pharmacokinetic model, transdermal drug delivery

## Abstract

**Purpose:**

The development of a new two-dimensional (2D) model to predict follicular permeation, with integration into a recently reported multi-scale model of transdermal permeation is presented.

**Methods:**

The follicular pathway is modelled by diffusion in sebum. The mass transfer and partition properties of solutes in lipid, corneocytes, viable dermis, dermis and systemic circulation are calculated as reported previously [Pharm Res 33 (2016) 1602]. The mass transfer and partition properties in sebum are collected from existing literature. None of the model input parameters was fit to the clinical data with which the model prediction is compared.

**Results:**

The integrated model has been applied to predict the published clinical data of transdermal permeation of caffeine. The relative importance of the follicular pathway is analysed. Good agreement of the model prediction with the clinical data has been obtained. The simulation confirms that for caffeine the follicular route is important; the maximum bioavailable concentration of caffeine in systemic circulation with open hair follicles is predicted to be 20% higher than that when hair follicles are blocked.

**Conclusions:**

The follicular pathway contributes to not only short time fast penetration, but also the overall systemic bioavailability. With such *in silico* model, useful information can be obtained for caffeine disposition and localised delivery in lipid, corneocytes, viable dermis, dermis and the hair follicle. Such detailed information is difficult to obtain experimentally.

## Introduction

A major challenge for scientific research regarding skin penetration of drugs, cosmetics, etc., is the development of robust non-animal methods to test percutaneous absorption and bioavailability. Dermal absorption due to exposure to agrochemicals and environmental pollutants is also becoming a global concern. Experimental approaches including *in vivo, ex vivo, in vitro* and clinical studies on human volunteers are often expensive and time consuming (1,2). Additionally, there is a general trend in safety regulatory guidelines worldwide to move away from animal testing of cosmetic products and ingredients (e.g. the European Commission) (3). In parallel to the advancement in experimental methods, *in silico* modelling of dermal absorption and delivery has been demonstrated to be useful in refining and reducing the experiments needed, to enable faster design of new products and more reliable safety assessment, and to improve the understanding of the transport process (2). In addition, a mechanistic model can help analyse the relative importance of different penetration pathways; it can be used to examine the impact of physico-chemical properties of the chemical, the physiological variability and application scenarios on penetration (4). As a result, *in silico* modelling has become an important tool in the study of topical and transdermal delivery.

Within this context, quantitative structure-permeability relationship (QSPR) models emerged mainly focusing on estimating the permeability coefficient or maximum flux of the chemicals (5). The compartmental approach, which treats the skin layers as different units of uniform concentration (6), was the next step of *in silico* modelling. Then, the introduction of more sophisticated diffusion-based models has attracted substantial attention since its appearance. These models produce spatially explicit and time dependent predictions of transdermal permeation following topical exposure. The majority of early diffusion-based models do not consider the heterogeneity of the stratum corneum (6–10). When the bricks-and-mortar structure of the stratum corneum was introduced and its importance acknowledged, the main challenge was to obtain the transport properties of chemical compounds in different domains of the skin. In some cases the transport properties are obtained by fitting to experimental skin penetration data (11,12). In such cases the main problem is that the model only works well with that particular experimental dataset. This limits the range of chemicals the model can be used for prediction.

Subsequently, Wang *et al*. (13) created a predictive model of transdermal permeation that derived transport properties of the solute from fundamental principles. Chen *et al*. (14,15) presented a multi-scale approach using a similar bricks-and-mortar structure for the stratum corneum. These later modelling studies adopted a multi-scale approach where transport properties of skin lipids and cornecocytes are determined separately, e.g. through molecular modelling and QSPRs, achieving improved prediction accuracy. Later, Dancik *et al*. (16) and Chen *et al*. (17) further included viable epidermis and dermis. Some excellent review articles have been published to summarise recent progress in this area; see e.g. (4,6,18). Recently we extended the model of Chen *et al*. (17) to include absorption into the systemic circulation and subsequent kinetics (19).

However, as far as the penetration pathways are concerned there is a noticeable gap with respect to the follicular pathway. In the past decade many studies (20–29) confirmed and highlighted the important contribution of the hair follicles to transdermal penetration. Although hair follicles occupy only ca. 0.1% of the skin surface, their diffusion coefficient can be orders of magnitude higher than that in the stratum corneum, and thus the overall effect can be significant (30). This pathway has been considered in some simple compartmental models (21,31,32) which, however, have limited predictive capability because of the need for parameter fitting of the model to experimental data.

The aim of this study is to develop a mechanistic model of the follicular pathway and integrate the follicular pathway into our latest multi-scale model of transdermal permeation (19). The stratum corneum, viable epidermis and dermis as well as the systemic circulation kinetics are modelled using the same methodology as before (14,17,19). The follicular pathway was integrated by considering the physiological and compositional properties of sebum and hair. The model’s predictive capability is demonstrated through simulating an *in vivo* study of topical application of caffeine on skin with or without hair follicle blocking (22). Predicted caffeine plasma concentration-time profiles showed good agreement with the reported clinical data. A sensitivity analysis has been conducted to demonstrate the rational of the chosen sebum partition and transport properties of caffeine and the effect of variability in such properties on the overall transdermal delivery of caffeine.

## Material and Methods

This section describes the method for modelling the follicular route of dermal absorption, and its integration with the previously reported multi-scale model of transdermal permeation and bioavailability (19). The technical details concerning the partition and transport properties of chemicals in skin and the blood circulation kinetics can be found in the appendices.

### The Hair Follicle Route: Modelling Approach

The main focus of this study is on the follicular pathway. Although there exist debate with regard to the actual route of follicular penetration (20,29), studies have been conducted to examine at which phase of the hair growth cycle follicular penetration occurs. Domashenko *et al*. (33) reported a study on mouse skin and human scalp xenograft. They concluded that transfection of liposomes occurred only at the onset of a new growing stage of the hair cycle (33). In contrast another study, one that the assumptions of the current approach are based on, conducted on 8 human volunteers suggested that penetration through the shunt pathway only occurs when the hair follicle is active (27), where active hair follicle is characterised by hair growth and/or sebum production (34). It is also known that sebum is a penetrable medium (35–37) and that the diffusion coefficient in sebum (35,36) is usually several orders of magnitude faster than that in hair (38). The above, alongside with the fact that hair it is a very dense material mainly made of keratin, support the argument that the sebum is the main transport route of follicular pathway. The gap between the hair infundibulum and the skin is assumed to be filled with sebum.

**Fig. 1:**
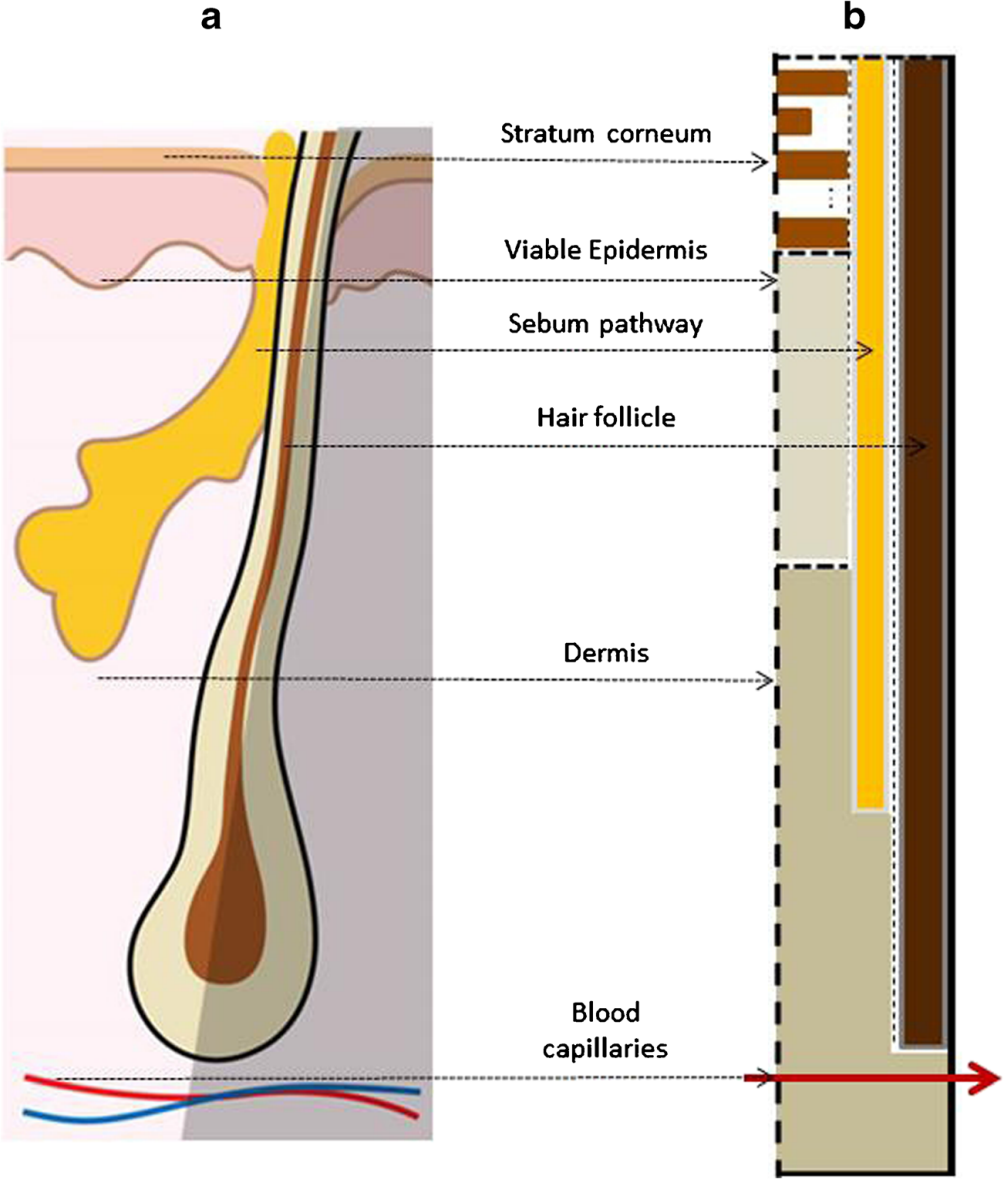
Simplification of hair follicle anatomy (**a**) into a 2D modelling domain (**b**). Note that the modelling domain corresponds to the unshaded area in (**a**).

Based on the above assumption, the hair follicle anatomy (Fig. 1a) is converted into a 2D domain of heterogeneous material as illustrated in Fig. 1b, upon which a mathematical model is developed. Figure 1b shows the different compartments considered in the computer model, where follicular penetration is simplified to be through the vertical sebum layer and the hair follicle itself is impermeable. In addition, the skin near a certain hair follicle is considered to be symmetric with respect to the hair follicle, and thus only half of the anatomy around hair (the unshaded area in Fig. 1a) needs to be modelled. The dimensions (and density with respect to skin surface area) of the hair follicle in this model are specific to body sites and can be set by the users, accounting for the variability from site to site on human body as reported in the literature (39,40).

Figure 1b also shows the other skin compartments around the hair follicle, which are needed for the integration of the follicular pathway with these compartments in modelling. The bricks-and-mortar structure of the stratum corneum can be seen at the top. Viable epidermis and dermis are modelled as homogeneous compartments; in the dermis compartment the systemic circulation is included as previously reported (19). Note that the sebaceous gland is not explicitly modelled. Further details regarding the integrated modelling framework are presented in the next section.

### The Integrated Mathematical Model

**Fig. 2:**
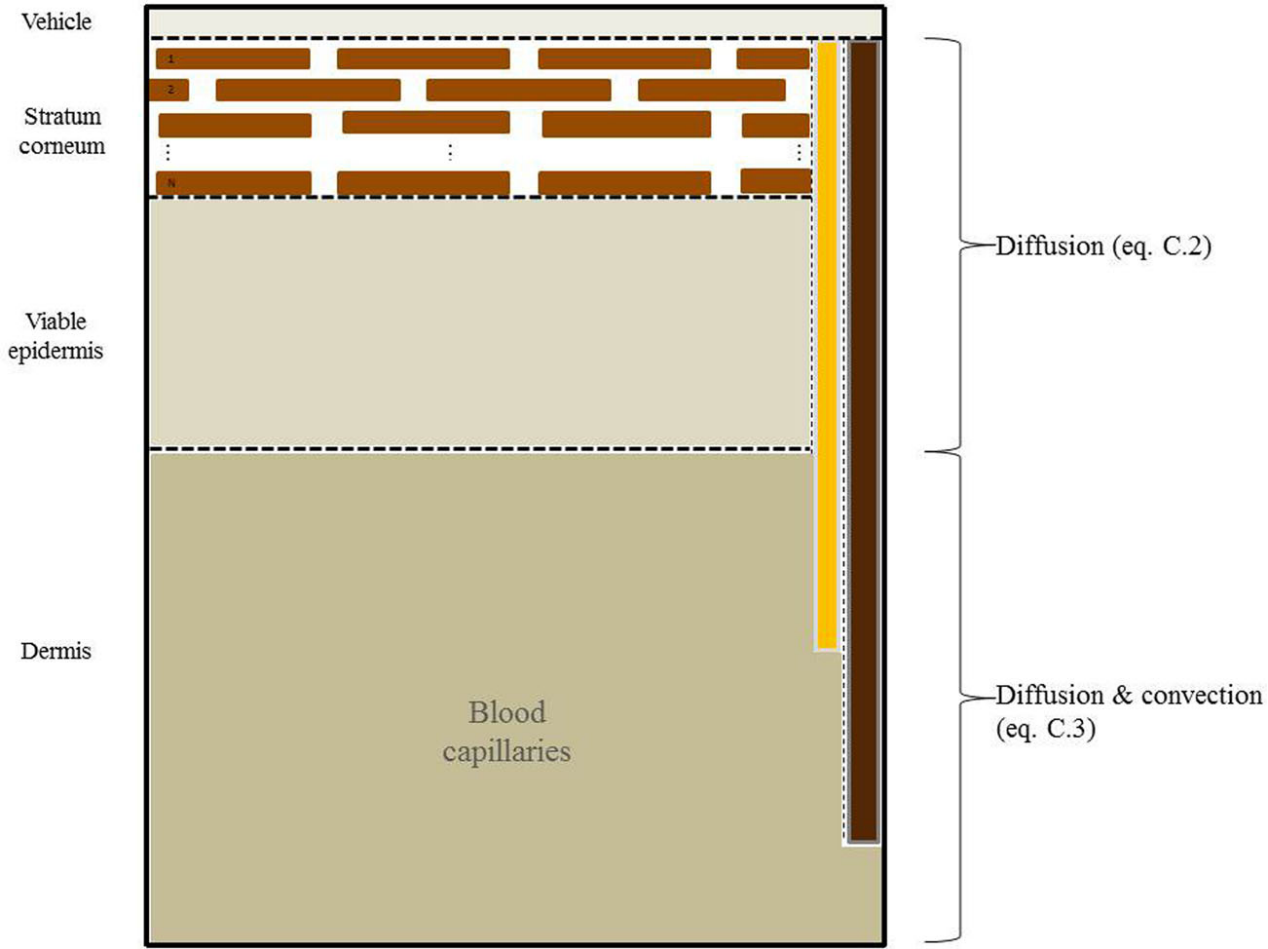
Model framework for transdermal permeation and systemic bioavailability including the three pathways through the stratum corneum: intercellular (through lipid), transcellular, and follicular. Note that the hair itself, illustrated as the rightmost *brown* vertical area, is assumed impermeable. Not to scale.

Figure 2 shows the complete modelling framework where the hair follicle, shown on the right hand side of the schematic, is integrated with the rest of skin compartments. Briefly, on the top is a homogenous vehicle layer, followed by the bricks-and-mortar structure of the stratum corneum (bricks: corneocytes; mortar: lipid), where the number of corneocyte layers, *N*, is specific to body site. Further down are the homogeneous viable epidermis and dermis, and the blood capillaries in the dermis where the solute clearance into systemic circulation is calculated. With the full thickness skin, hair and blood capillary considered, this framework intends to model the transdermal permeation and kinetics *in vivo*. Simplifications compared to the real hair follicle arrangement have been made. The bending of the stratum corneum in the follicular orifices as well as the funnel–shaped infundibulum (39) were initially considered. Separate simulations showed that including such detailed representation of the hair follicle region had an impact in the local region but negligible effect on the overall transdermal permeation. Although *in vivo* studies concerning the role of follicular pathway are less common than *in vitro* ones, there is a general uncertainty regarding the suitability of *in vitro* tests for predicting *in vivo* situations with respect to the contribution of the follicular pathway to transdermal permeation mainly due to pre-processing of the samples (41–43). Therefore, the focus of this study is to model the *in vivo* situations.

The governing equations include the diffusion equation that describes the transport of solute in all compartments as illustrated in Fig. 2. Inside dermis, mass transfer due to convection in blood is also included. Mass transfer calculation between the interfaces of compartments (e.g. between lipid and corneocyte in the stratum corneum, or between sebum and dermis) requires the partition coefficients of the solutes, as detailed in Appendix A and B. At the four boundaries of the entire modelling domain (solid lines in Fig. 2), zero flux is assumed. To solve these partial differential equations, in which the concentration of the solute changes with both spatial coordinates and time, the 2D domain in Fig. 2 is discretised into rectangular grids (19). Within each of the grids, the partial differential equation is converted to an ordinary differential equation (ODE) (Appendix C) using the standard method of lines (19). Due to constraint on space, detailed gridding is not presented, though it suffices to state that a large number of grids (1880 in total) are used to ensure that the results are independent of numerical inaccuracy due to this discretisation. The model was implemented in C++ with ODEs solved by using the CVODE solver, a part of the SUNDIALS computational package (44).

The partition and diffusion coefficients in various compartments, except those for sebum, are obtained from the QSPR models calculated from the physicochemical properties of the solute and skin structures; these models have been published in the literature and are collated in Appendix A and B. With the lack of reliable QSPR models for sebum, the experimentally measured partition and diffusion coefficients were used and will be discussed subsequently.

The vehicle of topical application is an important compartment that needs to be properly modelled, especially for *in vivo* finite-dose applications. In some cases (as in the caffeine case described below), the applied dose results in the concentration of the chemical in the vehicle exceeding its solubility in the corresponding solvent. The excess portion over solubility is modelled as solids. The rate of diffusion of the chemical from vehicle into skin is generally slower than the dissolution rate of solids in the vehicle. Therefore the vehicle is assumed to remain saturated until the excessive amount is fully dissolved (to make up the depletion of vehicle due to absorption into skin). The vehicle is then switched to a finite source in the model.

### Model Demonstration

As demonstration, the model was applied to simulate the reported clinical study (22) of topically applied caffeine to human chest. In this experiment an ethanol and propylene glycol (30:70, *v*/v) solution containing 2.5% caffeine was applied to the chest of six healthy volunteers in two different set-ups: before and after blocking of the hair follicles with a wax-mixture. The application area was 25 cm^2^ with a dose of 2 mg cm^−2^. The solution was left to evaporate, and since ethanol is known to evaporate within minutes, in the simulation the vehicle was simplified to consist of only propylene glycol and caffeine. During the experiments, blood samples were taken after each application at different times. The measured concentration of caffeine in the plasma with and without follicular blocking was reported to show the contribution of the follicular pathway to the overall transdermal penetration. This plasma concentration profile will be compared with model predictions.

**Fig. 3:**
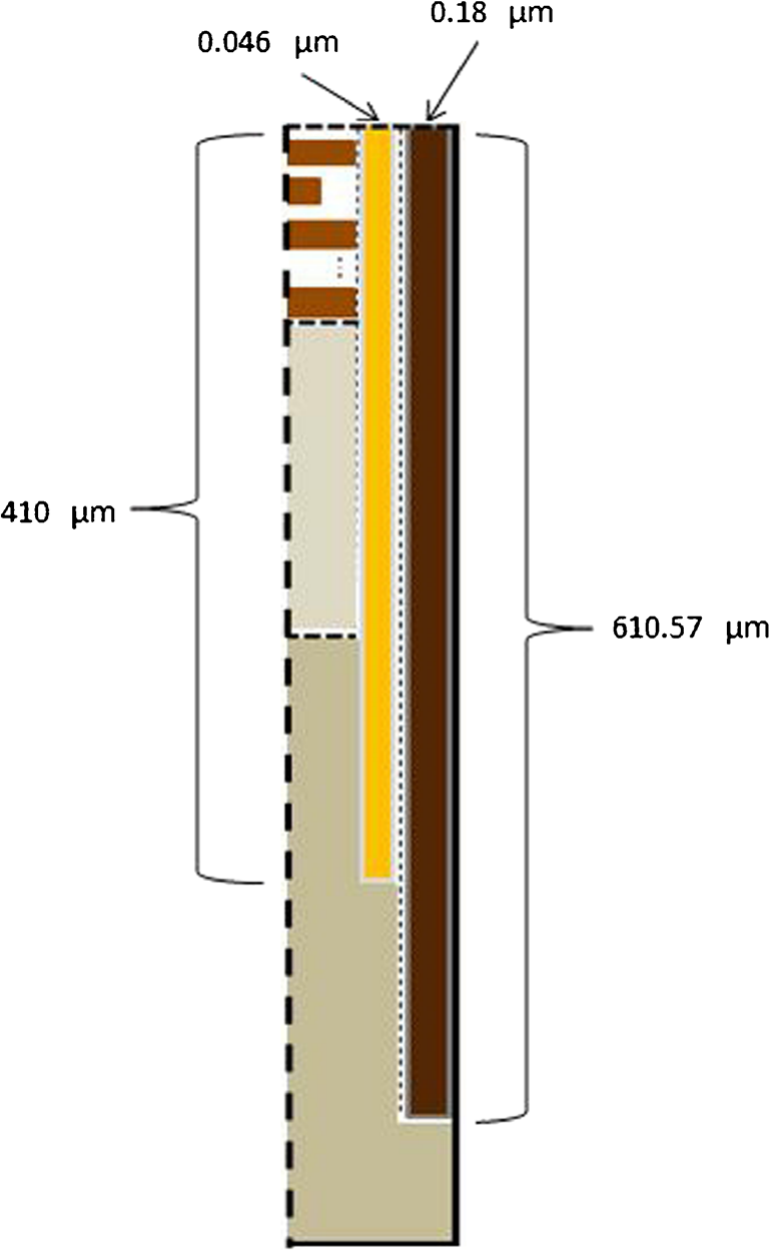
Hair follicle region geometry presenting the width of hair and sebum layers. The numbers presented in the schematic are subjected to scaling. Detailed information is given in the text.

The input parameters used for simulation are listed in Table I, where the physicochemical properties listed are used to calculate the partition and diffusion coefficients as detailed in Appendix A and B. The dimensions of skin including the hair follicle are chest specific. Specifically, the stratum corneum thickness in chest was set to 14 μm (45). The viable epidermis thickness is set to 100 μm and dermis thickness 1000 μm (46). Regarding the follicular pathway, the vertical depth of the hair is set to be 610.57 μm (20). The vertical depth of the sebum is set to be 410 μm which is approximately the depth of the sebaceous glands in the skin (47). The radius of hair in the thorax was reported to be ca. 40 μm (48) and that of the hair follicle opening 50 μm (49). This suggests that the lateral width of the sebum layer in Fig. 1b is 10 μm, by assuming that sebum completely fills the space between hair and the follicular opening. Furthermore, Otberg *et al*. (49) showed that the average area of follicular orifices in thorax is 0.19% of the skin surface. To directly represent this ratio using the actual diameter of hair follicle with a computer model would require a large simulation domain and computational power. Here, the width of the stratum corneum (and the viable epidermis and dermis beneath) is kept to three corneocytes width (i.e. 120 μm) to save computation expenses. Accordingly the width of sebum is scaled down to 0.046 μm to meet the above ratio. (Detailed calculation is as follows. Given the lateral width of stratum corneum of 120 μm and the surface area ratio of 0.19%, the entire follicle opening radius is 0.19 %  × 120/(1 − 0.19%) = 0.228 μm. Furthermore, it was found that the radius of hair in chest is ca. 40 μm (48) and that of the hair follicle opening 50 μm (49), suggesting the sebum annulus radius is 1/5 of the entire follicle opening. Therefore in the scaled geometry the sebum annulus width is determined to be 0.228/5 = 0.046 μm.) The dimensions of the hair follicle are summarised in Fig. 3.

**Table I:** Model Input Parameters

	Parameter	Value
Caffeine	Molecular weight	194.1906 Da
	Octanol-water partition coefficient	0.85
	Solubility in propylene glycol	28.9 kg m^−3^
	Fraction of non-ionised solute	0.99
	Fraction of unbound solute to albumin	0.63
Vehicle	Thickness	0.1912 μm
	Initial caffeine concentration	37.35 kg m^−3^
Stratum corneum	Thickness	14 μm
	Number of corneocyte layers	16 (chest)
	Width of corneocytes	40 μm
	Height of corneocytes	0.8 μm
	Thickness of intercellular lipid	0.075 μm
	Lateral spacing between corneocytes	0.075 μm
Viable epidermis	Thickness	100 μm
Dermis	Thickness	1000 μm
Hair follicle	Depth of HF	610.57 μm
Sebum	Sebum tube width	0.046 μm*
	Sebum tube height	410 μm
Blood	Cardiac output	5.6 L min^−1^
	Skin blood flow as fraction of cardiac output	5%
	Caffeine clearance in blood	0.078 L h^−1^ kg^−1^
Subject	Weight	70 kg
	Application area	25 cm^2^

^*^Value scaled down to match the reported ratio of surface area of hair follicle to that of skin; see text for details

The partition and diffusion coefficients of caffeine in various skin compartments (except in sebum) were obtained from the established QSPR equations detailed in Appendix A and B, using the physicochemical properties of caffeine given in Table I. The partition and diffusion coefficients in sebum were reported for some chemicals but the QSPR models developed had substantial uncertainty in the prediction (35,36). In the present study, the diffusion coefficient was set to be the same as the measured data of butyl 4-hydroxybenzonate (9.67×10^−11^ m^2^ s^−1^, (36)), which has the same molecular weight as caffeine; according to Mitragotri (50) the diffusion coefficient in a certain media is primarily determined by the molecular weight of the chemical. The sebum:water partition coefficient of caffeine was obtained from a standard equilibrium experiment conducted at the China Agricultural University (private communications). The clearance rate of caffeine in systemic circulation was based on the reported data for oral delivery 0.078 L h^−1^ kg^−1^ (51). The vehicle:water partition coefficient is estimated to be 0.87 from the solubility of caffeine in the vehicle over that in water (16). The diffusion coefficient of caffeine in the vehicle was estimated to be 9.16×10^−10^ m^2^ s^−1^ using the Stokes-Einstein equation (Eq. B.1). Table II summarises the diffusion and partition properties of caffeine in different compartments.

**Table II:** Diffusion and Partition Properties of Caffeine in Different Compartments

Skin compartment	Diffusion coefficient (m^2^s^−1^)	Partition coefficient with respect to water
Vehicle	9.16×10^−10^	0.87
Lipid	7.62×10^−12^	0.80
Corneocytes	3.95×10^−15^	2.56
Sebum	9.67×10^−11^	0.06
Viable epidermis & Dermis	1.85×10^−10^	0.84

## Results and Discussion

**Fig. 4:**
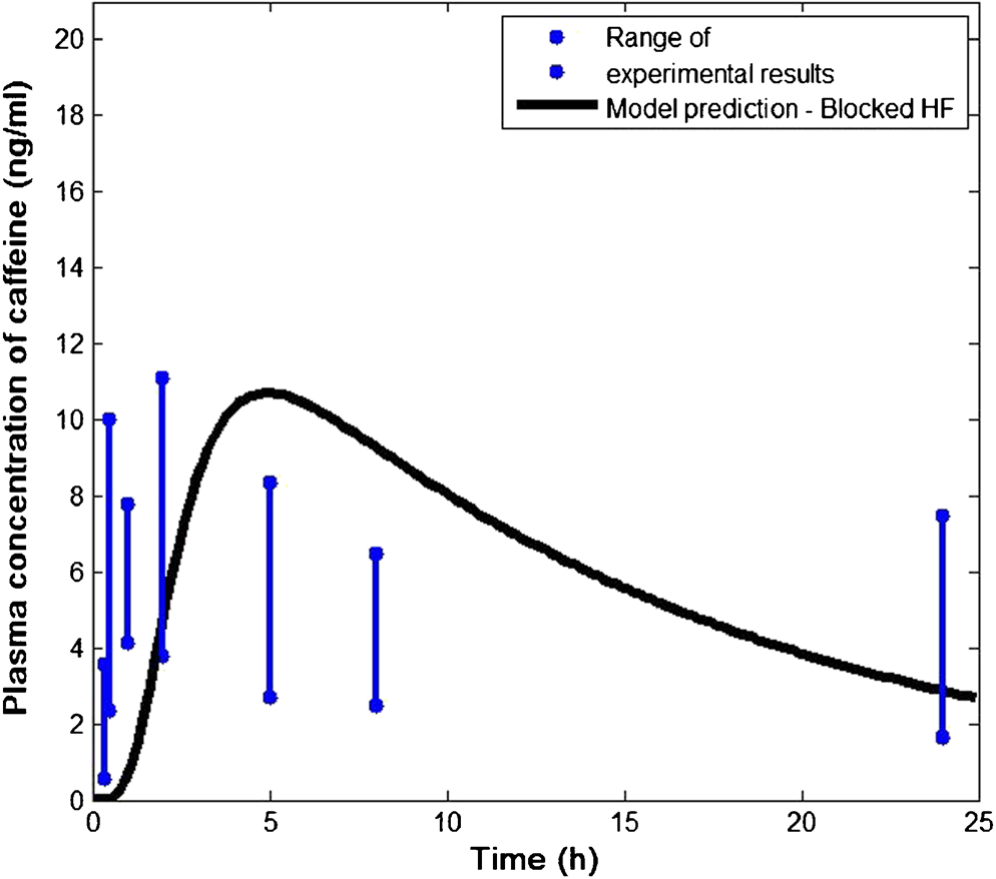
Comparison of predicted plasma concentration of caffeine with published clinical data. *In vivo* data were obtained with blood samples taken from six human volunteers with blocked hair follicles (HF) (22).

**Fig. 5:**
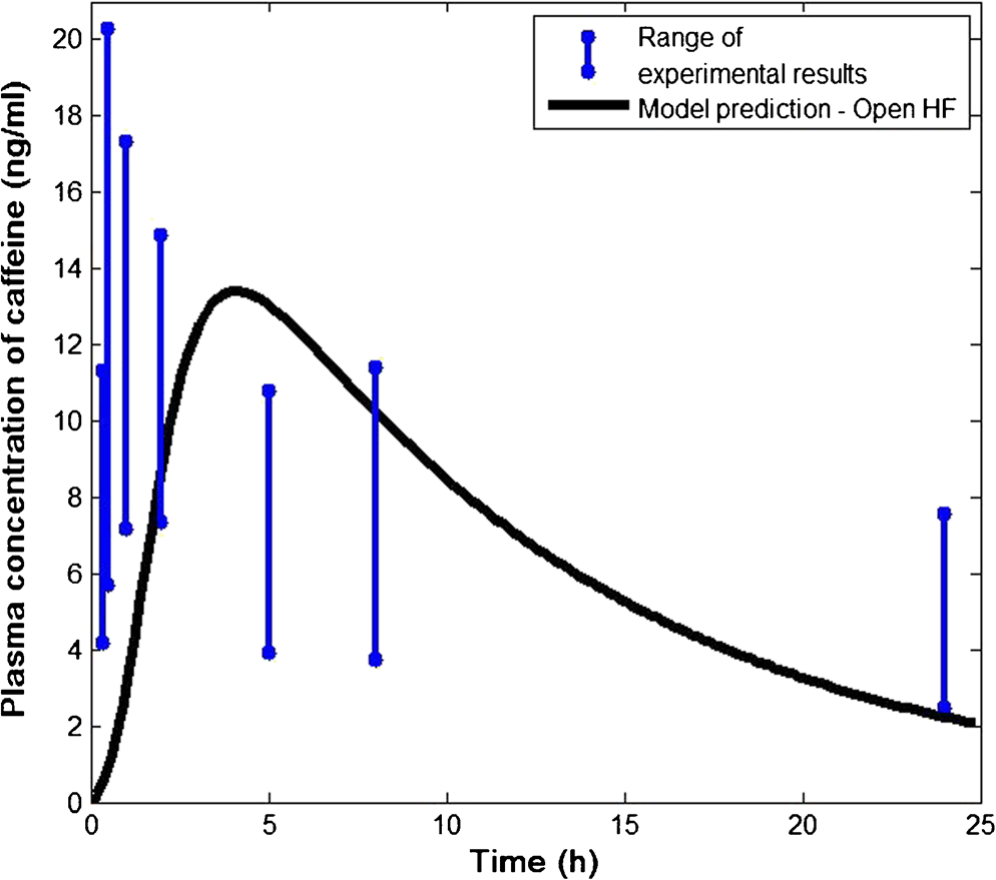
Comparison of predicted plasma concentration of caffeine with published clinical data. *In vivo* data were obtained with blood samples taken from six human volunteers with open hair follicles (HF) (22).

The predicted plasma concentration of caffeine is shown in Fig. 4 (blocked hair follicle) and Fig. 5 (open hair follicle) in comparison with the published experimental data (22). The range of the concentrations was obtained from six subjects reflecting significant inter-subject variability. In both cases, the model prediction appears to be in good agreement with the *in vivo* data.

**Fig. 6:**
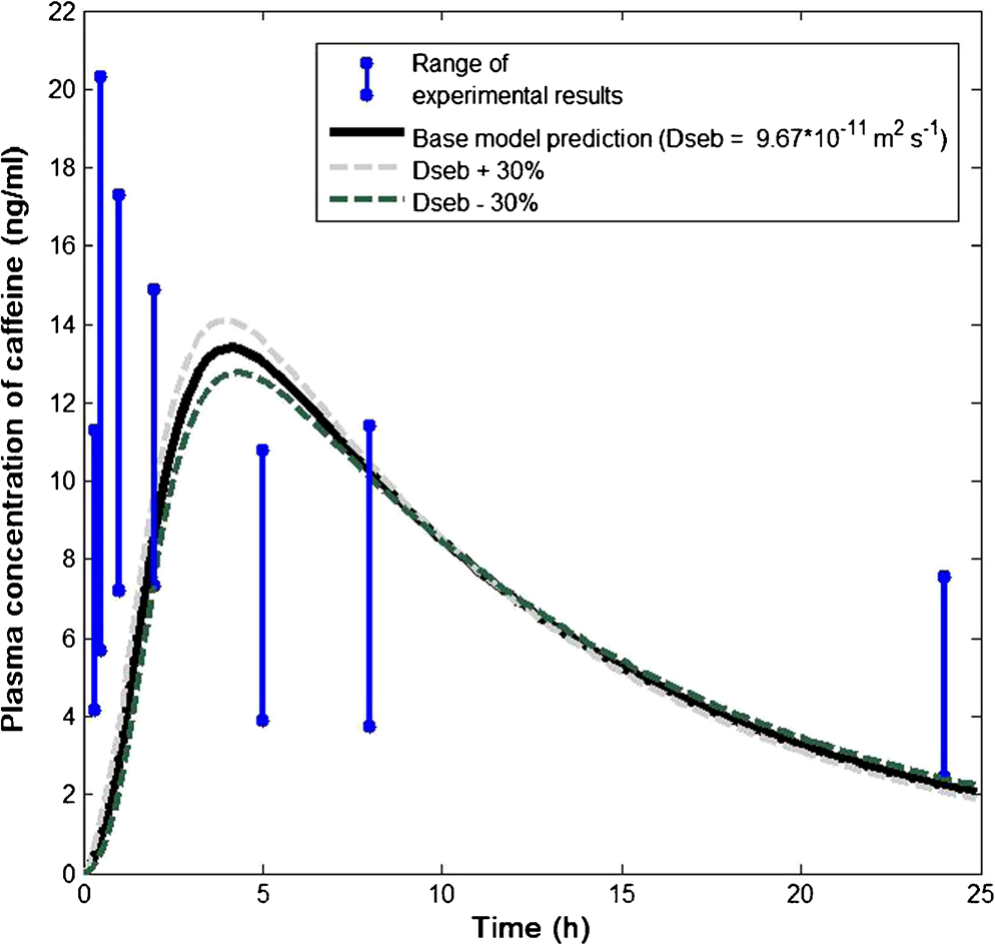
Sensitivity analysis with respect to the diffusion coefficient of caffeine in sebum

Subsequently, sensitivity analysis was conducted to explore the impact of the parameter variability relating to sebum on model predictions. Sensitivity analysis respect to other skin compartments has been reported elsewhere (e.g. (19)). Figure 6 shows the predicted systemic kinetics when the diffusion coefficient of caffeine in sebum is subjected to 30% variability. Clearly, decreasing (increasing) the diffusion coefficient results in slower (faster) penetration of caffeine into the blood, since the penetration through the follicular pathway becomes slower (faster). A similar effect was observed when subjecting the partition coefficient and sebum width to similar extent of variability (results not reported).

**Fig. 7:**
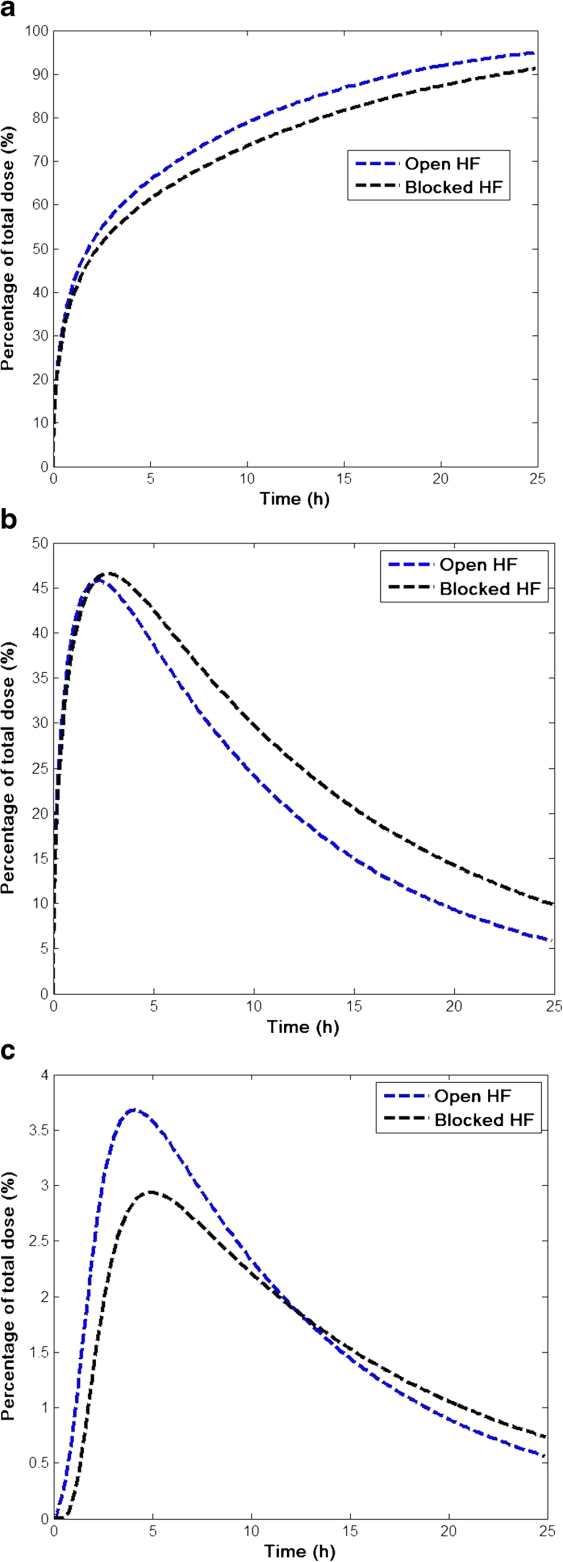
Predicted kinetics of transdermal delivery of caffeine with open and blocked hair follicles. (**a**) Caffeine permeated into the skin (**b**) caffeine disposited in the skin and (**c**) caffeine delivered to systemic circulation.

Figure 7 illustrates the predicted kinetics of caffeine absorbed from the vehicle (Fig. 7a), disposition in skin (Fig. 7b, all skin compartments except blood), and delivery into systemic circulation (Fig. 7c), in terms of the percentage of total dose applied for open and blocked hair follicles. Caffeine is mildly hydrophilic and thus its partitioning into oily sebum is substantially less than partitioning into either lipid or corneocytes in stratum corneum (c.f. Table II). However, the diffusion coefficient in sebum is 10+ times higher than that in lipid (and several orders of magnitude higher than in corneocytes). As a result, the overall effect of the follicular pathway is significant. It can be seen from Fig. 7a that when hair follicles are open, a greater and faster uptake of caffeine by the skin is observed. Figure 7b shows that, due to the additional follicular pathway that bypasses the stratum corneum to reach viable epidermis and dermis, caffeine resides for less time in the skin with faster and higher delivery to the blood (Fig. 7c), when compared with the situation where hair follicles are blocked.

In Table III the relative contribution of the follicular pathway to caffeine delivery to systemic circulation, as predicted by the model, is quantified in terms of the maximum plasma concentration (C_max_), the time to reach C_max_ (T_max_) and the area under curve (AUC). AUC represents the overall systemic bioavailability of dermal exposure for a given time. These values clearly show that hair follicles contribute significantly to the overall transdermal permeation especially at the early stage of application. Specifically, the AUC in systemic circulation one hour after application is substantially higher when hair follicles are open (0.86 ng h mL^−1^) then when hair follicles are blocked (0.1 ng h mL^−1^), with a percentage difference of 88%. Even at 10 h post application, the percentage difference of AUC between blocked and open hair follicles is still very significant, at 21%. The difference in C_max_ and T_max_ is also significant. It is worth noting that the predicted difference also agreed to a large extent with the clinical study (22). These results highlight the importance of hair follicles for the bioavailability in the skin and systemic circulation after dermal exposure to caffeine.

**Table III:** Predicted Systemic Kinetics Following Topical Delivery of Caffeine with Open and Blocked Hair Follicles (HF). Percentage Change = (Open HF – Blocked HF)/(Open HF)

	C_max_ (ng mL^−1^)	T_max_ (h)	AUC (1 h) (ng h mL^−1^)	AUC (10 h) (ng h mL^−1^)
Open HF	13.40	4.0	0.86	97
Blocked HF	10.7	5.0	0.10	77
Percentage change	20%	−25%	88%	21%

**Fig. 8:**
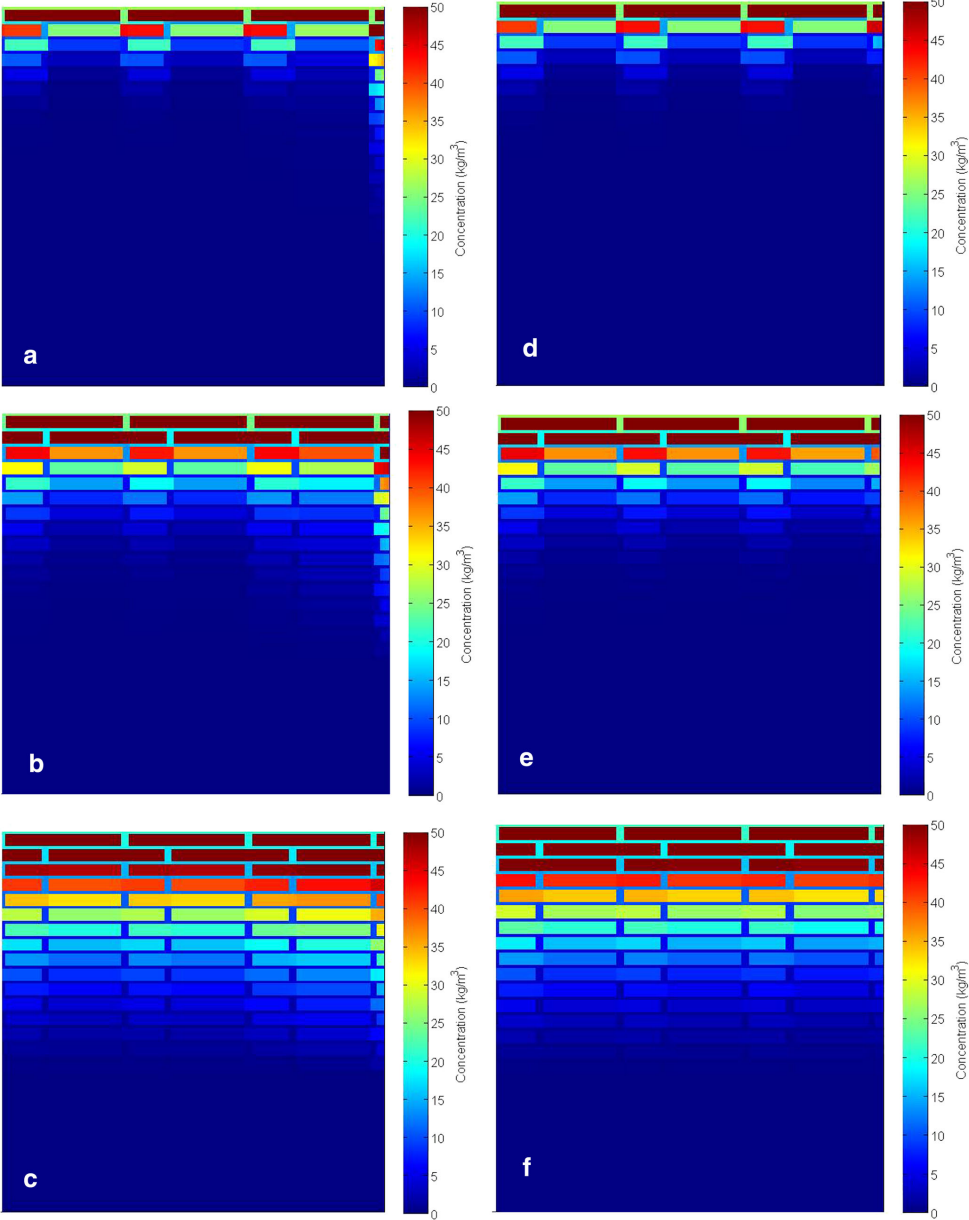
Subcellular disposition of caffeine in the stratum corneum of the skin with open (**a**, **b**, **c**) and blocked (**d**, **e**, **f**) hair follicles (HF) at 5 min (**a**, **d**), 20 min (**b**, **e**) and 1 h (**c**, **f**) after application.

Figure 8 presents the detailed 2D disposition of caffeine in the stratum corneum predicted by the model at different time steps after the application of caffeine. The two sections of the figure represent the simulations with the follicular pathway open (a-c) and blocked (d-e). The concentration profiles clearly show the contribution of the follicular pathway in the permeation process. Caffeine concentration in the corneocytes is visibly higher than in the lipid and this is due to the high level of binding of caffeine in corneocytes compared to any other compartment (Table II). At early times (e.g. up to 20 min), the concentration profiles are noticeably different with the follicular route being of significant importance in penetration.

**Fig. 9:**
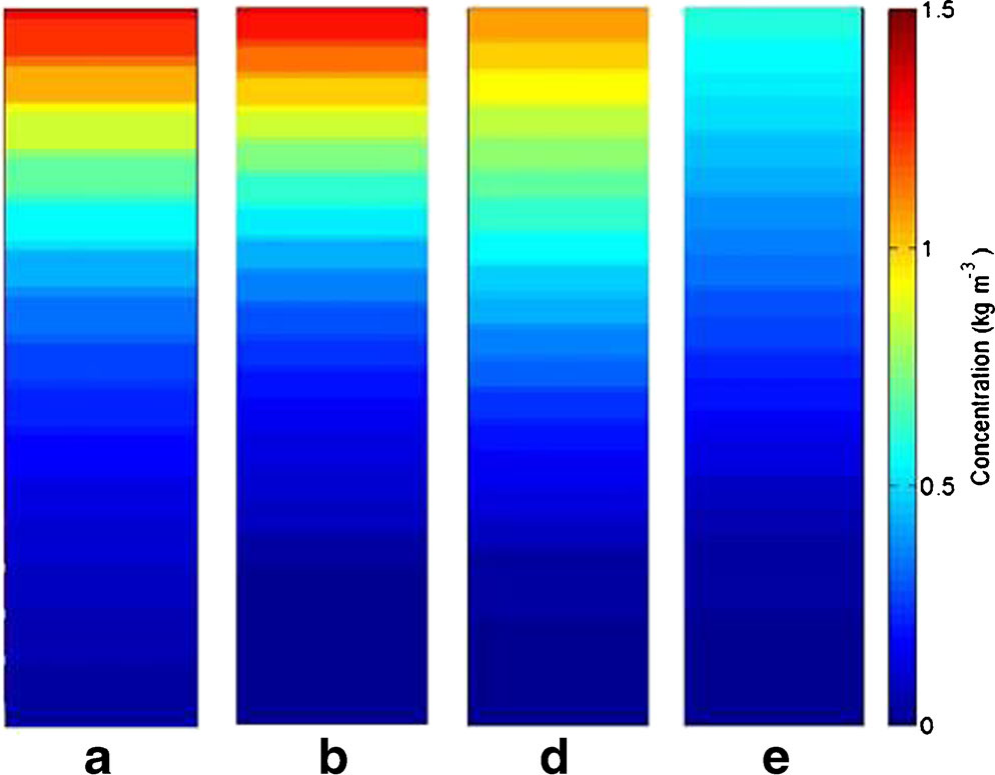
Disposition of caffeine in the sebum layer when hair follicles are open; (**a**) *t* = 5 min; (**b**) *t* = 20 min; (**c**) *t* = 1 h; (**d**) *t* = 10 h.

Due to the relatively low partition coefficient in sebum, the disposition of caffeine in sebum is not apparent in Fig. 8a-c. In Fig. 9, the concentration profile in sebum is rescaled and shown for different times. As can be seen from this figure, high concentrations are observed during the early stage after application whereas as time proceeds, caffeine concentration in sebum decreases. This, together with the overall penetration profile illustrated in Figs. 5, 6, and 7, suggests that the impact of follicular pathway on caffeine delivery is more significant at the early times after application. This observation from model prediction agrees with experimental studies in the literature, e.g. (21,22)

## Conclusions

This paper presents a new *in silico* model for transdermal permeation and systemic bioavailability with the integration of the follicular pathway. The multi-scale model considers the important molecular and microscopic principles involved in skin permeation and systemic absorption. To our knowledge, this is the first model in the open literature that has the capability to offer quantitative prediction of the three major pathways (intercellular, intracellular and follicular) of transdermal permeation. The model confirms the importance of the follicular pathway. Prediction of the disposition of chemicals in various skin compartments enhances our understanding of the local pharmaco−/toxico-kinetics after skin exposure for assessing efficacy and toxicity. This model could provide improved *in silico* screening for pharmaceuticals and industrial chemicals and be a valuable tool in extrapolating from *in vitro* experiments to *in vivo* exposure conditions - a key component to reduce the reliance on animal models. It should be noted that the model is applicable to topical application of small molecules (<500 Da) on a given body site, provided that the appropriate physiological parameters, physicochemical properties of the solute and vehicle properties are specified. Further validation of the model with experimental studies of more compounds and/or exposure scenarios is needed. Currently, a more comprehensive validation of the developed model against advanced imaging data is being planned.

## APPENDIX A: Physical properties

These properties are required in the QSPR models in Appendix B to predict partition and diffusion coefficients in various compartments of skin, and mass transfer into blood.

### A.1. Solute radius

The radius of the solute (Å) is estimated using the equation below based on the molecular weight (MW) of the solute (52):

A.1 $$ {\mathrm{r}}_{\mathrm{s}}=\sqrt[3]{3/4\pi \times 0.9087\kern0.2em \mathrm{MW}} $$

### A.2. Volume fraction of water in stratum corneum at saturation

Swelling and shrinking of the stratum corneum (SC) is not considered in this study (14) thus the porosities of both SC lipids and corneocytes are considered to be constant. Here the porosity is defined as the volume fraction of the SC pores at fully dehydrated state. The volume fraction of water in the SC at saturation (φ_sc_) is related to the volume of lipid (V_l_), keratin (V_k_), and saturated water (V_w_), content by φ_sc_ = V_w_/(V_w_ + V_l_+V_k_). The water phase distributed into the SC lipids and corneocytes (V_w_= V_wl_ +V_wk_) with the corresponding saturated volume fraction of water (or porosity) in SC lipids and corneocytes as φ_m_= V_wl_/ (V_wl_ +V_l_) and φ_b_= V_wk_/(V_wk_ +V_k_). Since V_w_ + V_l_ + V_k_= V_wl_ + V_l_ + V_wk_ +V_k_, it follows that V_w_/φ_sc_ = V_wl_/φ_m_ +V_wk_/φ_b_. Using the relationship V = m/ρ (where m is the mass and ρ is the density), it follows that porosity of the SC is related to the porosities of SC lipids and corneocytes by (14):

A.2 $$ \frac{\raisebox{1ex}{${\mathrm{f}}_{\mathrm{l}}$}\!\left/ \!\raisebox{-1ex}{${\uprho}_{\mathrm{l}}$}\right.+\raisebox{1ex}{${\mathrm{f}}_{\mathrm{k}}$}\!\left/ \!\raisebox{-1ex}{${\uprho}_{\mathrm{k}}$}\right.}{1-{\phi}_{\mathrm{sc}}}=\frac{\raisebox{1ex}{${\mathrm{f}}_{\mathrm{l}}$}\!\left/ \!\raisebox{-1ex}{${\uprho}_{\mathrm{l}}$}\right.}{1-{\phi}_{\mathrm{m}}}+\frac{\raisebox{1ex}{${\mathrm{f}}_{\mathrm{k}}$}\!\left/ \!\raisebox{-1ex}{${\uprho}_{\mathrm{k}}$}\right.}{1-{\phi}_{\mathrm{b}}} $$

where f_l_ (≈12.5%) and f_k_(≈87.5%) are the dry mass fractions of SC lipid and keratin. The overall porosity of the SC can be related to its saturated water content (mass fraction) by the following equation:

A.3 $$ {\phi}_{\mathrm{sc}}=\frac{{\mathrm{f}}_{\mathrm{sc}}}{\uprho_{\mathrm{w}}\left(1-{\mathrm{f}}_{\mathrm{sc}}\right)\left({\mathrm{f}}_{\mathrm{l}}/{\uprho}_{\mathrm{l}}+{\mathrm{f}}_{\mathrm{k}}/{\uprho}_{\mathrm{k}}\right)+{\mathrm{f}}_{\mathrm{sc}}\ } $$

In order to estimate the above parameters, the user needs to enter the thickness of the SC. The dimensions and offset of bricks and mortars in the SC are fixed according to our previous study (14) .

### A.3. The hydraulic permeability of the medium (k)

The hydraulic permeability is estimated from the correlation derived from Jackson and James (53) given as:

Α.4 $$ \mathrm{k}=\upbeta {\mathrm{r}}_{\mathrm{f}}^2\ {\left(1-{\uptheta}_{\mathrm{b}}\right)}^{\upgamma} $$

where β and γ are fitting parameters that will be explained later on and θ_b_ is the actual fraction of water in the corneocyte phase. r_f_ is the radius ok keratin microfibril (r_f_ = 3.5 nm (54))

### A.4 Volumetric blood flow

The volumetric blood flow (Q) can be estimated according to physiology. It is known that the average resting cardiac output is ca. 5.6 L min^−1^ for a human male and 4.9 L min^−1^ for a female (55). The overall blood flow to skin is estimated to be 5% of the cardiac output (56). Therefore for a male and female respectively the equations used are shown below:

## APPENDIX B: Diffusion and partition properties

### B.1 Vehicle properties

Diffusion coefficient in vehicle and any other medium can be calculated using the Stokes-Einstein equation (57):

B.1 $$ {\mathrm{D}}_{\mathrm{w}}=\frac{\mathrm{KT}}{6\uppi \upeta {\mathrm{r}}_{\mathrm{s}}} $$

where K is the Boltzmann constant, T is the room temperature (309 K), η is the viscosity of the medium and r_s_ is the solute radius as calculated in Eq. (A.1).

Partition coefficient in the vehicle is calculated using the solubility of the chemical in water and the solubility of the chemical in the vehicle. If the vehicle is water then it equals 1.

### B.2. Lipid properties in stratum corneum

Diffusion coefficient in the SC lipid is related to the solute radius (A.1) by the equation proposed by Mitragotri (50):

B.2 $$ {\mathrm{D}}_{\mathrm{m}}=\left\{\begin{array}{c}2\times {10}^{-9} \exp \left(-0.46\ {\mathrm{r}}_{\mathrm{s}}^2\right),\kern2.25em \mathrm{MW}\le 380\ \mathrm{Da}\\ {}\kern0.5em 3\times {10}^{-13},\kern8em \mathrm{MW}>380\  Da\ \end{array}\right. $$

The partition coefficient of lipid to water can be estimated using the equation below (17):

B.3 $$ {\mathrm{K}}_{\mathrm{mw}}=\frac{\uprho_{\mathrm{l}}}{\uprho_{\mathrm{w}}}{\mathrm{K}}_{\mathrm{ow}}^{0.69} $$

Where ρ_l_ = 0.9 g/cm^3^.

### B.3. Corneocyte properties in stratum corneum

Solute diffusion coefficient in the SC corneocytes (D_b_) is estimated in the same way as our previous study (14):

B.4 $$ {\mathrm{D}}_{\mathrm{b}}=\frac{ \exp \left(-\upalpha {\mathrm{S}}^{\uplambda}\right)}{1+\frac{{\mathrm{r}}_{\mathrm{s}}}{\sqrt{\mathrm{k}}} + \frac{{\mathrm{r}}_{\mathrm{s}}^2}{3\mathrm{k}}}\times {\mathrm{D}}_{\mathrm{w}} $$

where α, β, γ and λ are model fitting parameters (α = 9.47, β = 9.32×10^−8^, γ = −1,17 and λ = 1,09), S = (1 - θ_b_)${\left(\frac{{\mathrm{r}}_{\mathrm{s}}+{\mathrm{r}}_{\mathrm{f}}}{{\mathrm{r}}_{\mathrm{f}}}\right)}^2$, k is the hydraulic permeability (Eq. (A.3)) and D_w_ the diffusion coefficient in water (Eq. (B.2)). The fitting parameters α, β, γ and λ were chosen after applying the above QSPR model to fit steady-state skin permeability data in the respective studies cited above.

The partition coefficient of corneocyte to water is estimated using the following equation (14):

B.5 $$ {\mathrm{K}}_{\mathrm{b}\mathrm{w}}=\left(1-{\upphi}_{\mathrm{b}}\right){\mathrm{K}}_{\mathrm{kw}}+{\uptheta}_{\mathrm{b}} $$

where K_kw_ is the solute binding constant to SC keratin and is estimated using the following reported eq. (58):

B.6 $$ {\mathrm{K}}_{\mathrm{k}\mathrm{w}}=\frac{\uprho_{\mathrm{k}}}{\uprho_{\mathrm{w}}}\times 4.2\ {\mathrm{K}}_{\mathrm{ow}}^{0.31} $$

where ρ_k_ = 1.37 g/cm^3^ and ρ_w_ = 1 g/cm^3^. In order to get this coefficient the user is required to enter the Octanol water partition coefficient of the chemical to be tested.

### B.4. Viable epidermis and dermis properties

As literature suggests that viable epidermis and dermis have similar multiphase compositions (16,59) the partition and diffusion properties in these two layers are assumed to be similar. The partition coefficient is calculated from the following equation proposed by Kretsos *et al*. in 2008 (59) and then modified by Ibrahim *et al*. in 2012 (60).

B.7 $$ {\mathrm{K}}_{\mathrm{v}}=0.7\times \left(0.68+\frac{0.32}{{\mathrm{f}}_{\mathrm{u}}}+0.025\ {\mathrm{f}}_{\mathrm{non}}{{\mathrm{K}}_{\mathrm{ow}}}^{0.7}\right) $$

where the three terms 0.68, 0.32/f_u_ and 0.025 f_non_ account for the chemical disposition in albumin accessible aqueous phase. f_non_ is the fraction of solute non-ionized in the aqueous phase and f_u_ is the fraction of unbound (to albumin) solute.

The diffusion coefficient is also based on the binding/partitioning properties of the dermis and is given from the equation below:

B.8 $$ {\mathrm{D}}_{\mathrm{v}}=\frac{10^{-8.15-0.655 \log \mathrm{MW}}}{0.68+\frac{0.32}{{\mathrm{f}}_{\mathrm{u}}}+0.025\ {\mathrm{f}}_{\mathrm{non}}{\mathrm{K}}_{\mathrm{mw}}} $$

## APPENDIX C: Overall simulation approach

The mass transfer equation is solved by a finite difference scheme. In order to do so the skin is divided into a finite number of smaller elements.

The mass transferred between neighbouring elements A and B is given by (14):

C.1 $$ {\kern1.5em \mathrm{q}}_{\mathrm{A}\mathrm{B}}=\frac{{\mathrm{A}}_{\mathrm{i}}}{\frac{\updelta_{\mathrm{A}}}{{\mathrm{D}}_{\mathrm{A}}}+\frac{{\mathrm{K}}_{\mathrm{A}\mathrm{B}}{\updelta}_{\mathrm{B}}\ }{{\mathrm{D}}_{\mathrm{B}}}}\left({\mathrm{C}}_{\mathrm{A}}-{\mathrm{K}}_{\mathrm{A}\mathrm{B}}{\mathrm{C}}_{\mathrm{B}}\right) $$

where q_AB_ is the flux of solute from grid A to grid B, A_i_ is the interfacial area between the two grids, δ_A_ and δ_B_ are the corresponding diffusion length, D_A_ and D_B_ are the diffusion coefficients of the two elements, K_AB_is the solute partition coefficient between the two elements (K_AB_ = 1 if the two grids are the same element and K_AB_ = K_Aw_/K_Bw_ if element A is different from B), C_A_ and C_B_ are the solute concentrations in the two elements.

According to mass conservation principles, concentration of each element A satisfies the following equation:

C.2 $$ \frac{{\mathrm{dC}}_{\mathrm{A}}}{{\mathrm{dt}}_{\mathrm{s}}}=-\frac{\sum {\mathrm{q}}_{\mathrm{A}\mathrm{B}}}{\mathrm{V}} $$

where C_A_, is the solute concentration in element A, V is the element volume, t_s_ is the time, q_AB_ is the flux of solute into/out of element A from neighbouring element B.

When it comes to dermis in addition to diffusion, convection needs to be considered when modelling the transport between dermis and the capillaries due to blood flow. The full explanation of this approach can be found in the respective reference (19). For a dermis grid I, the mass balance equation is:

C.3 $$ {\mathrm{V}}_{\mathrm{A}}\frac{\mathrm{d}{\mathrm{C}}_{\mathrm{A}}}{\mathrm{d}\mathrm{t}}=-\sum {\mathrm{q}}_{\mathrm{A}\mathrm{B}}+{\mathrm{Q}}_{\mathrm{b},\mathrm{A}}\left({\mathrm{C}}_{\mathrm{b}}-\frac{{\mathrm{C}}_{\mathrm{A}}}{{\mathrm{K}}_{\mathrm{d}\mathrm{b}}}\right) $$

where Q_b , A_ is the volumetric blood flow into dermis grid A, C_b_ is the solute concentration in the blood and K_db_ is the partition coefficient from dermis to blood.

The systemic circulation and clearance is described by the following equation:

C.4 $$ {\mathrm{V}}_{\mathrm{b}}\frac{{\mathrm{dC}}_{\mathrm{b}}}{\mathrm{dt}}=\mathrm{N} \ast \sum \left[\ {\mathrm{Q}}_{\mathrm{b},\mathrm{A}} \ast \left(\ \frac{{\mathrm{C}}_{\mathrm{A}}}{{\mathrm{K}}_{\mathrm{db}}}-{\mathrm{C}}_{\mathrm{b}}\right)\ \right]\ {-\mathrm{KC}}_{\mathrm{b}} $$

where V_b_ is the volume of the whole body blood vessel, and KC_b_is the first order clearance that may include transport into other tissues and metabolism.

## Abbreviations

CVODE: C-language variable-coefficients ODE solver
HF: Hair follicle
ODE: Ordinary differential equation
QSPR: Quantitative structure-property relationship
SC: Stratum corneum
SUNDIALS: Suite of non-linear and differential/algebraic equation solvers
VE: Viable epidermis
